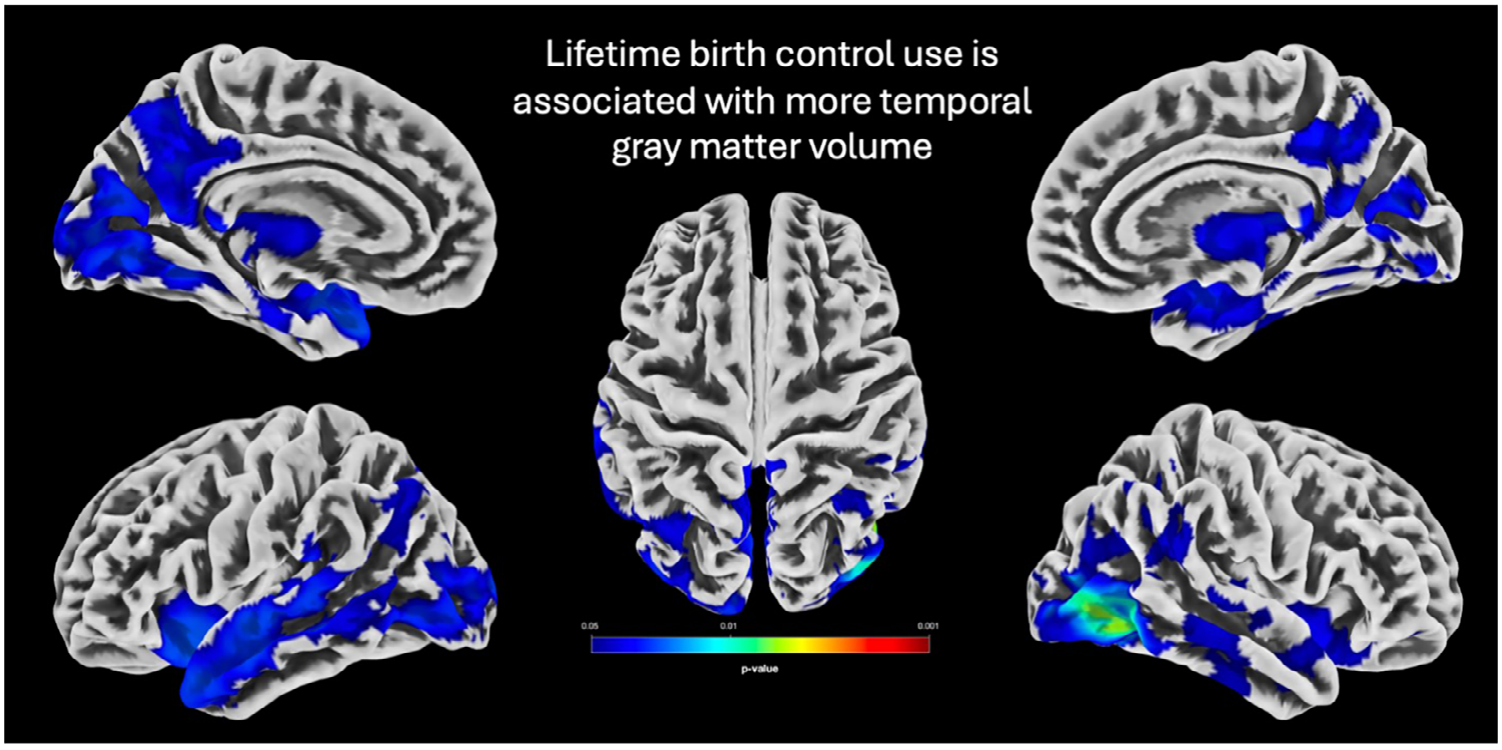# Lifetime Exposure to Estrogen Preserves Brain Health in Older Women in the IGNITE study

**DOI:** 10.1002/alz70861_108310

**Published:** 2025-12-23

**Authors:** Robyn A Honea, Amber Watts, Ankit Patel, William Guiler, Shannon Donofry, Hayley Ripperger, Sarah L Aghjayan, Chaeryon Kang, Lauren Oberlin, Swathi Gujral, George Grove, Haiqing Huang, Brad Sutton, Jeffrey M. Burns, Eric D Vidoni, Arthur F. Kramer, Edward McAuley, Charles Hillman, Anna Marsland, M. Ilyas Kamboh, Kirk I. Erickson

**Affiliations:** ^1^ University of Kansas Alzheimer's Disease Research Center, Fairway, KS USA; ^2^ University of Kansas Medical Center, Kansas City, KS USA; ^3^ University of Kansas, Lawrence, KS USA; ^4^ University of Kansas School of Medicine, Kansas City, KS USA; ^5^ Brain Aging and Cognitive Health Lab, Psychology, University of Pittsburgh, Pittsburgh, PA USA; ^6^ University of Pittsburgh, Pittsburgh, PA USA; ^7^ Department of Psychology, University of Pittsburgh, Pittsburgh, PA USA; ^8^ AdventHealth Neuroscience Institute, Orlando, FL USA; ^9^ University of Pittsburgh School of Medicine, Pittsburgh, PA USA; ^10^ AdventHealth Research Institute, Neuroscience, Orlando, FL USA; ^11^ University of Illinois, Urbana, IL USA; ^12^ University of Kansas Alzheimer's Disease Research Center, Kansas City, KS USA; ^13^ University of Kansas Medical Center, Alzheimer's Disease Research Center, Fairway, KS USA; ^14^ Beckman Institute, University of Illinois, Urbana, IL USA; ^15^ Center for Cognitive & Brain Health, Northeastern University, Boston, MA USA; ^16^ Northeastern University, Boston, MA USA; ^17^ University of Pittsburgh Alzheimer's Disease Research Center (ADRC), Pittsburgh, PA USA; ^18^ AdventHealth Research Institute, Orlando, FL USA

## Abstract

**Background:**

Two‐thirds of AD patients are women, and aging‐related hormonal changes may contribute to these sex differences. Lower lifetime exposure to estrogen, a neuroprotectant, is associated with AD‐related brain atrophy. Estrogen‐based therapies are associated with better brain health, including preserved brain volume in areas that atrophy with aging. We investigated whether hormone‐based medications (birth control (BC), menopausal hormone therapy (MHT)) were associated with structural neuroimaging markers of aging and Alzheimer’s disease. We hypothesized that timing of hormone‐based therapies would be associated with larger brain volume and greater cortical thickness in older women in temporo‐limbic regions.

**Method:**

We investigated baseline associations in Investigating Gains in Neurocognition in an Intervention Trial of Exercise (IGNITE), a 12‐month, multi‐site, randomized aerobic exercise trial in 459 female participants aged 65‐80. We used SPM12 and CAT12 software to process 3D‐T1 MPRAGE images, and conducted voxel‐based (VBM‐gray matter volume) and surface‐based morphometry (SBM‐thickness) to investigate voxel‐wise differences between individuals that had a history of a) BC use vs. no BC use; b) MHT use vs. no MHT use and c) MHT use (no BC) vs. no MHT use (no BC), accounting for age, education, study site, and *APOE4* carrier status.

**Result:**

In the VBM analysis, BC use (average start age 22) was associated with larger left inferior frontal gyrus volume (cluster k=2613, FWE corrected *p* ‐value= 0.004, Figure 1). BC timing (starting age, duration) did not add significantly to the models. In the SBM analysis, combined BC and MHT use was associated with a thicker left middle temporal cortex (vertices = 496, FWE corrected *p* ‐value=0.019). Interestingly, when looking at MHT use without the use of BC earlier in life, there were still brain benefits of increased cortical thickness in the right lingual gyrus (vertices=459, FWE corrected *p* ‐value=0.032).

**Conclusion:**

Our results support prior evidence that lifetime estrogen exposure is important for healthy brain aging, particularly with regard to brain volume. These findings have clinical implications for estrogen‐based therapies throughout the lifespan. Understanding sex differences in AD is critical for developing sex‐specific prevention strategies and treatments as part of a precision medicine approach.